# Effects of 5G-modulated 3.5 GHz radiofrequency field exposures on HSF1, RAS, ERK, and PML activation in live fibroblasts and keratinocytes cells

**DOI:** 10.1038/s41598-023-35397-w

**Published:** 2023-05-23

**Authors:** Alexandre Joushomme, Rosa Orlacchio, Lorenza Patrignoni, Anne Canovi, Yann Loïck Chappe, Florence Poulletier De Gannes, Annabelle Hurtier, André Garenne, Isabelle Lagroye, François Moisan, Muriel Cario, Philippe Lévêque, Delia Arnaud-Cormos, Yann Percherancier

**Affiliations:** 1 Bordeaux University, CNRS, IMS laboratory, UMR5218, F-33400 Talence, France; 2 Limoges University, CNRS, XLIM, UMR 7252, F-87000 Limoges, France; 3 Institut Universitaire de France (IUF), F-75005 Paris, France; 4 Paris Sciences et Lettres Research University, F-75006 Paris, France; 5 Bordeaux University, INSERM, BMGIC Laboratory, UMR1035, F-33000 Bordeaux, France

**Keywords:** Environmental impact, Fluorescent proteins

## Abstract

The potential health risks of exposure to radiofrequency electromagnetic fields from mobile communications technologies have raised societal concerns. Guidelines have been set to protect the population (e.g. non-specific heating above 1 °C under exposure to radiofrequency fields), but questions remain regarding the potential biological effects of non-thermal exposures. With the advent of the fifth generation (5G) of mobile communication, assessing whether exposure to this new signal induces a cellular stress response is one of the mandatory steps on the roadmap for a safe deployment and health risk evaluation. Using the BRET (Bioluminescence Resonance Energy-Transfer) technique, we assessed whether continuous or intermittent (5 min ON/ 10 min OFF) exposure of live human keratinocytes and fibroblasts cells to 5G 3.5 GHz signals at specific absorption rate (SAR) up to 4 W/kg for 24 h impact basal or chemically-induced activity of Heat Shock Factor (HSF), RAt Sarcoma virus (RAS) and Extracellular signal-Regulated Kinases (ERK) kinases, and Promyelocytic Leukemia Protein (PML), that are all molecular pathways involved in environmental cell-stress responses. The main results are (i), a decrease of the HSF1 basal BRET signal when fibroblasts cells were exposed at the lower SARs tested (0.25 and 1 W/kg), but not at the highest one (4 W/kg), and (ii) a slight decrease of As_2_O_3_ maximal efficacy to trigger PML SUMOylation when fibroblasts cells, but not keratinocytes, were continuously exposed to the 5G RF-EMF signal. Nevertheless, given the inconsistency of these effects in terms of impacted cell type, effective SAR, exposure mode, and molecular cell stress response, we concluded that our study show no conclusive evidence that molecular effects can arise when skin cells are exposed to the 5G RF-EMF alone or with a chemical stressor.

## Introduction

Within the fast deployment of mobile telecommunications over the last decades, the 5th generation (5G) of wireless networks was designed to improve on the 4G LTE technology by resolving issues linked with the exponential usage increase, the number of connected devices, and the need for higher reliability and lower latency^1,2^. Such achievements required new frequency bands in addition to those already deployed for the 2G, 3G, and 4G. Among them, the 3.4–3.8 GHz band offers a good trade-off between broadband coverage and speed, while the 26 GHz band, characterized by poor propagation and penetration inside buildings, will be deployed at a second stage to cover limited areas with high data traffic. Therefore, the 3.5 GHz band (also known as the C-band), which can use the same cell sites as the current 2.6 GHz and 1.8 GHz mobile antennas, is the core band of the current 5G.

The biological and health effects of exposure to environmental radiofrequency electromagnetic fields (RF-EMF) have been the subject of numerous studies since the late twentieth century, and are still the focus of societal concerns. This research field was also strengthened by the International Agency for Research on Cancer (IARC) decision in May 2011 to classify RF-EMFs as 2B carcinogens.

Notably, while the energy of RF photons is not strong enough to trigger chemical modifications in biological targets, such as DNA breaks, living tissue dielectric heating under RF-EMF exposure is fully characterized. Guidelines were therefore established to protect the population against the associated risks^3^. However, whether RF-EMF exposure can trigger “nonthermal” effects (i.e. biological effects not caused by temperature elevation in living tissue) remains a difficult-to-study issue. Since there is no mechanistic support for these effects, the scientific community can only rely on empirical research concerning potential RF-EMF nonthermal effects^4–6^.

Unfortunately, very few data from scientific studies are available on the potential biological hazard presented by RF-EMF exposure to the new frequency signals used in 5G. To the best of our knowledge, only six articles, performed by four different teams, have already been published that explored the effects of unmodulated or GSM-modulated 3.5 GHz RF-EMF on living matter, but no biological studies have since been published with 5G-modulated 3.5 GHz RF-EMF signals. Dasgupta et al. exposed developing zebrafish to unmodulated 3.5 GHz RF-EMF at a specific absorption rate (SAR) of 8.27 W/kg for 6 to 48 h post-fertilization under isothermal culture conditions^7,8^. These authors measured subtle but persistent sensorimotor effects and a modest transcriptomic disruption at 48 h post-fertilization, with 28 differentially expressed genes in the exposed groups. Whether these effects are still measurable using SAR levels complying with the guidelines remains to be determined. Wang et al. assessed the impact of short- and long-term exposures using unmodulated 3.5 GHz RF-EMF at three different SAR (2.6, 26, and 260 mW/kg) on Drosophila melanogaster^9,10^. These authors showed a moderate but significant impact on insect activity, sleep, and development that was accompanied by a modification in the expression of genes involved in circadian rhythms, thermal stress, oxidative stress, and humoral immunity. Yang et al. assessed the effect of unmodulated 3.5 GHz RF-EMF exposure with a SAR of 0, 2, 4, or 10 W/kg for 72 h on anxiety-like behavior and the auditory cortex (ACx) in guinea pigs. This study pointed to a SAR-dependent increase in oxidative stress, apoptosis induction, and ultrastructural damage in ACx with no increase in animals’ anxiety and hearing thresholds^11^. Finally, Bektas et al. assessed whether GSM-modulated 3.5 GHz signal induced a change in energy homeostasis and redox balance in the brains of diabetic and healthy rats exposed for 2 h a day for 30 days at a calculated SAR of 0.323 W/kg in the brain^12^. After RF-EMF exposure, among diabetic and healthy rats, decreased total antioxidant levels and increased total oxidant and H_2_O_2_ levels were observed in brain tissues. These authors also observed that RF-EMF caused the variation in hormone levels that influence food intake and energy metabolism in the brain and increased the number of degenerated neurons in the hippocampus. Altogether, these studies suggest a potential effect of 3.5 GHz RF-EMF on several stress response pathways in eucaryotes organisms.

Assessing whether new RF-EMF technologies induce a cellular stress response under well-controlled exposure conditions is thus one of the mandatory steps on the roadmap for health risk evaluation of 5G signals. In particular, at the molecular level, whether Heat-Shock Proteins (HSP) expression and RAS/MAPK signal transduction pathways are induced has been highly debated due to the central role played by these molecular systems stress responses of cells^13,14^. Questions also remain regarding the occurrence of oxidative stress in RF-EMF exposed cells^15^. Therefore, it is crucial to further assess the effect of newly deployed RF signals, such as the 5G signal, on generic molecular mechanisms involved in cellular stress response. In the present study, we investigated the effect of a 5G-modulated 3.5 GHz RF-EMF signal on basal and chemically-induced Heat-Shock Factor 1 (HSF1), RAt Sarcoma virus (RAS) and Extracellular signal-Regulated Kinases (ERK) kinases, and ProMyelocytic Leukemia (PML) activities.

HSF1 is the “master regulator” of heat-shock proteins’ transcription in eukaryotes^16^. RAS and ERK kinases are key elements of Ser/Thr mitogen-activated protein-kinase (MAPK) cascades that relay extracellular signals to intracellular processes following the activation of RAS^17,18^. The RAS/MAPK signaling pathway is pivotal in regulating various cellular processes such as gene expression, cellular growth, and survival. Finally, PML protein is the keystone of the formation of PML Nuclear bodies (PML NBs). They are spherical nuclear domains that form in response to various stress conditions, including oxidative stress, and that are of prime importance for apoptosis, senescence, DNA repair, epigenetic control, as well as control of oncogenesis^19^. PML NBs formation is therefore a marker of cellular stress responses and is also important for the activity of numerous transcription factors and nuclear proteins, including HSF1 and ERK^20–24^. Our study used human skin fibroblasts and keratinocytes as cellular models as skin cells will become the primary target tissue of 5G exposure to consider for risk assessment^25^. These cells were continuously or intermittently (5 min ON/10 min OFF) exposed to the 5G-modulated 3.5 GHz RF-EMF signal at 0.25, 1, and 4 W/kg under isothermal conditions for 24 h before HSF1, RAS, ERK, and PML activities were assessed, thanks to well-worn Bioluminescence Resonance Energy Transfer (BRET)-based molecular probes^13,14,26^.

## Material and methods

### Plasmids

RAS and ERK BRET sensors were developed by replacing the fluorescent energy donor (ECFP or Turquoise-GL) and the fluorescent energy acceptor (YPet-M) of the EKAREV and RaichuEV-ras FRET probes^27^ with nanoLuciferase (nLuc^28^) and mNeonGreen (mNeonG^29^), respectively. Mammalian expression vector coding ERK (pEKAREV) and RAS (pRaichuEV-Ras) FRET probes were kindly provided by Dr Matsuda M (Kyoto University, Japan). The cDNA coding for nLuc and mNeonG were first synthetized (Genescript, Rijswijk, The Netherland) and then cloned in place of the fluorescent energy donor, between *N*ot*I* and *X*ba*I,* in pEKAREV and pRaichuEV-Ras expression vectors.

The cDNA encoding the nLuc-HSF1 and mNeonG-HSF1 proteins were derived from rLuc-HSF1 and sYFP2-HSF1 expression vectors^13^, in which Renilla Luciferase II and sYFP2 groups were replaced by nLuc and mNeonG, respectively, between BamHI and EcoRI. The HSP90 expression vector was also described in Poque et al.^13^.

Similarly, the cDNA encoding the nLuc-PMLIII and mNeonG-SUMO1 proteins were derived from rLuc-PMLIII and YFP-SUMO1 expression vectors^26^, in which Renilla Luciferase and YFP were replaced by nLuc and mNeonG, respectively, between BamHI and EcoRI.

### Reagents

As_2_O_3_ (A1010, 330 mM stock solution resuspended in 1 N NaOH) and MG132 (C2211, Z-Leu-Leu-Leu-al, 50 mM stock solution resuspended in DMSO) were from Sigma (Lyon, France). Phorbol-12-myristate-13-acetate (PMA) was acquired from Tocris (Bristol, UK), and Coelenterazine H from Nanolight Technology (Pinetop, AZ, USA).

### Cell culture and transfections

We have used the SV-40 immortalized skin fibroblast line established from a 19-year-old female with xeroderma pigmentosum (XP), complementation group D, the XP6BE line^30^ supplied by the Coriell Institute (Camden, NJ). XP6BE fibroblasts were maintained in Dulbecco's modified Eagle's medium – high Glucose (DMEM) (D6429, Sigma) supplemented with 10% fetal bovine serum, 100 units mL^-1^ penicillin and streptomycin. HaCaT keratinocytes^31^ were maintained in Keratinocyte-SFM (Ref 17005-034; Thermo Fisher Scientific, Waltham, MA, USA) supplemented with Bovine Pituitary Extract (BPE; Catalogue Number 13028-014, Gibco, Thermo Fisher Scientific, Waltham, MA, USA) & Human Recombinant EGF (catalog number 10450-13, Gibco, Thermo Fisher Scientific, Waltham, MA, USA) supplied with the kit Gibco™ Keratinocyte-SFM Supplement (Ref 37000015, Gibco, Thermo Fisher Scientific, Waltham, MA, USA). Twenty-four hours before transfection, cells were seeded at a density of 500,000 cells per well in 6-well dishes. Transient transfections were performed using polyethylenimine (PEI, linear, MW 25,000; catalogue number 23966 Polysciences, Inc., Warrington, PA, USA) with a PEI:DNA ratio of 4:1, as previously described^32^. For measurement of HSF1 activity, 0.1 µg of nLuc-HSF1 expression vector was co-transfected with 1.4 µg of mNeonG-HSF1 and 0.5 µg of HSP90 expression vectors. For measurement of RAS and ERK activities, 1 µg of pEKAREV or pRaichuEV-Ras BRET expression vectors were co-transfected with 1 µg of empty vector. For measurement of PML activity, 0.1 µg of nLuc-PMLIII expression vector was co-transfected with 1.9 µg of mNeonG-SUMO1. After overnight incubation, transfected cells were then detached, resuspended in DMEM w/o red phenol (Ref 21063-029, ThermoFisher scientific, Waltham, MA, USA) and replated at a density of 10^5^ cells per well in 96-well white plates with clear bottoms (Greiner Bio one, Courtaboeuf, France) pre-treated with d-polylysine (Sigma, Lyon, France) for reading with the Tristar2 luminometer (Berthold Technologies, Bad Wildbad, Germany) or onto 12 mm diameter glass coverslips (Knittel Glass, Braunschweig, Germany) treated with d-polylysine for the reading with the SpectraPro 2300i spectrometer (Acton Optics, Acton, MA, USA) (see below). Cells were left in culture for 24 h before being processed for the BRET assay.

### BRET measurements

**Figure 1:**
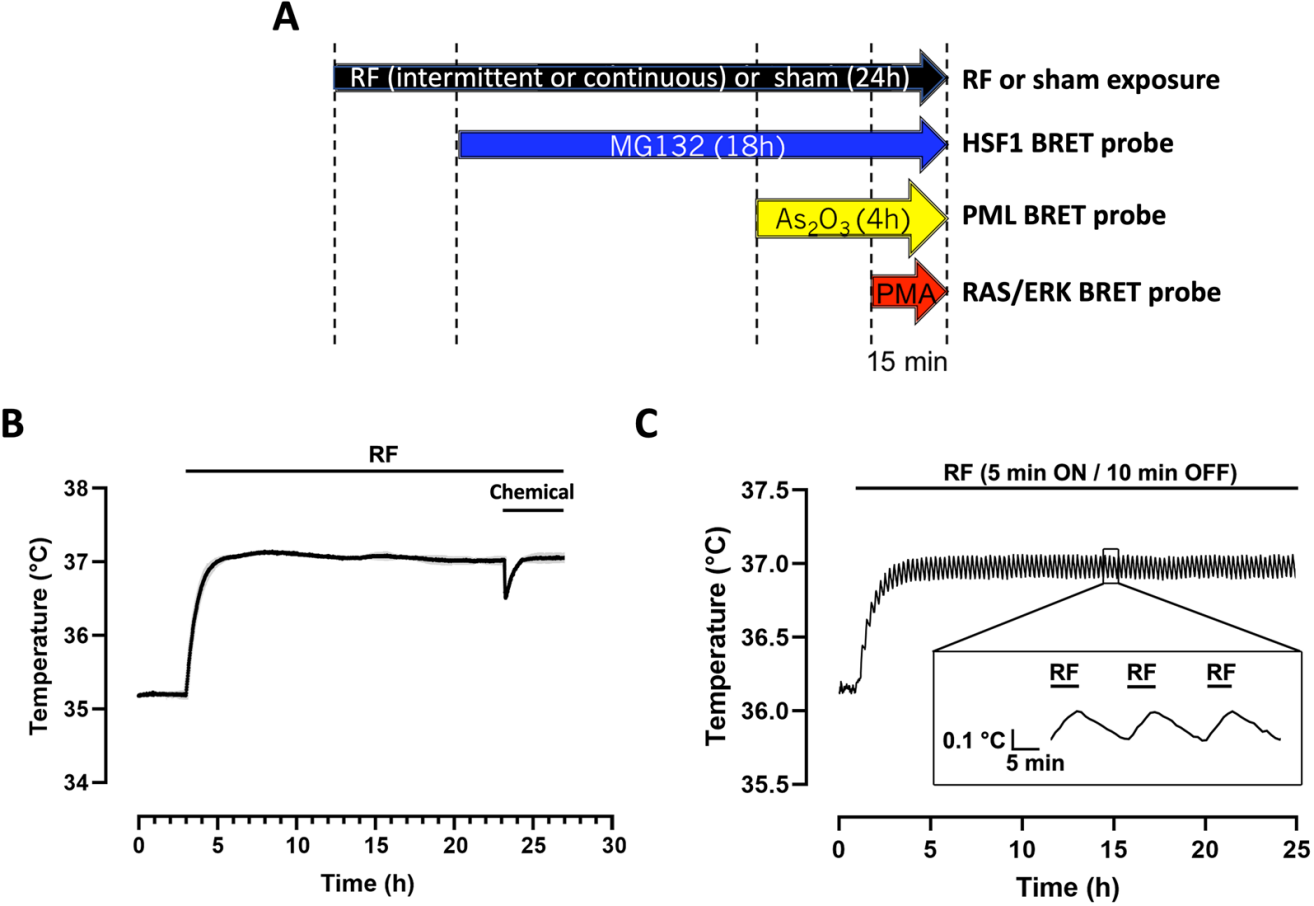
(**A**) Timeline of the RF-EMF exposure and chemical drugs addition in the cell culture medium. RF-EMF (or sham RF exposure) is applied for 24 h whatever the drug considered, that is injected for the indicated period. (**B**,**C**) Temperature variation in plates exposed to a continuous (**B**) or intermittent (**C**) 5G-modulated 3.5 GHz RF-EMF signal at 4W/kg. The temperature in the incubator was set to ensure cellular exposure at 37 °C and to compensate for the RF EMF-induced temperature increase at the onset of the exposure period. Drug injection induces a less than 0.5 °C transient drop in the cell culture temperature (**B**).

Cells were sham-exposed for 24 h (i.e. the cells were cultivated in absence of RF-EMF) or exposed to the indicated RF-EMF exposure conditions for 24 h. During the last 18 h, 4 h or 15 min of either sham or RF-exposure, the cells were incubated with the indicated concentration of MG132, As_2_O_3_ or PMA respectively (Fig. 1A). Only one chemical was tested in each 96-well plate; however various concentrations of the same chemical compound were tested simultaneously in a single plate by injecting 10X stock-solutions pre-heated at 37 °C using a multi channel pipette. When indicated, mock-treatment were performed by injecting only the solvating agent into the cell culture medium (DMSO for MG132 and PMA or water for As_2_O_3_).To perform the chemical treatment, the plates were removed from the reverberating chamber (see below “Cells exposure, exposure set-up, and dosimetry” section of the Material and methods) and immediately docked on a Thermostat Plus microplate Peltier heater (Eppendorf, Hamburg, Germany) to keep the cells at 37 °C. The sham-exposed plates were treated the same way. Removing the plate from the reverberating chamber, dispatching the chemicals at the various concentrations in the cell culture media using a multi-channel pipette and replacing the cells into the reverberating chamber took less than 1 min. After completion of the remaining RF exposure, 5 µM Coelenterazine H was added to the cell culture medium and the BRET signal was immediately acquired using a TriStar2 LB942 microplate reader (Berthold Technologies, Bad Wildbad, Germany) pre-heated at 37 °C and equipped with emission filters centered at 515 ± 20 nm for mNeonG (*I*_*acceptor*_) and 460 ± 20 nm for nLuc (*I*_*donor*_). Alternatively, for real-time BRET measurement under RF-EMF exposure, 5 µM Coelenterazine H was added to the cell culture medium 10 min before the end of the RF-EMF exposure and the full BRET spectra were recorded remotely for the last 5 min of RF-EMF exposure and the 5 next min in absence of RF-EMF. Full BRET spectra were acquired using an optical fiber linked to an IsoPlane SCT-320 Imaging Spectrograph equipped with a BLAZE:400B back illuminated CCD camera system camera for recording the full visible spectrum (Teledyne France—Princeton Instruments, Lisses, France).

The BRET signal was determined by calculating the ratio of the emission intensity measured in the acceptor window (*I*_*mNeonG*_) over the emission intensity measured in the donor window (*I*_*nLuc*_), according to Eq. (1)

1 $${\text{BRET}}=\frac{{I}_{\text{mNeonG}}}{{I}_{\text{nLuc}}}.$$

Due to the overlapping emission spectra of nLuc and mNeonG, a fraction of the light detected in the mNeonG filter originates from the luciferase emission, resulting in a contaminating signal^33^. In that configuration, the net BRET was therefore defined as the BRET ratio of cells co-expressing nLuc and mNeonG constructs minus the BRET ratio of cells expressing only the nLuc construct in the same experiment.

### BRET data processing and statistical analysis

The GraphPad Prism v8.00 for Mac software (GraphPad Software, La Jolla, CA, USA) was used for plotting dose–response curves and performing statistical analyses. The size of the error bars indicates the S.D. within the data set. Sigmoidal dose–response curves were fitted using Eq. (2):

2 $${\text{Y}}=\mathrm{Bottom}+\frac{(Top-Bottom)}{1+{10}^{logEC50-X}},$$

where X is the logarithm of agonist concentration and Y the response; Bottom is the Y value at the bottom plateau and is taken as the measure of basal level of activation of the various probes; Top is the Y value at the top plateau and Top–Bottom is taken as the measure of the maximal efficacy of a given chemical treatment on each BRET probe; Log EC50 is the X value when the response is halfway between Bottom and Top (Supp. Fig. 1). The EC50 value represents therefore a measure of the apparent potency of the various chemical compounds to trigger the activation of their cognate BRET probe (Supp. Fig. 1). Potencies of chemicals to activate or inhibit the different probes are expressed as pEC50 ± S.E.M (standard error of the mean), that is equal to –log EC50.

The one sample Wilcoxon signed-rank test was used to assess the statistical significance against the null hypothesis of the differences calculated in each independent experiment between sham (no RF EMF condition) and RF-EMF exposure conditions for basal BRET, chemicals’ potencies and efficacies (hereafter denominated ΔBasal BRET, ΔpEC50, and ΔMax efficacy, respectively). The total number of independent experiment (n) performed for each experimental condition is indicated. One sham exposure was performed for each RF-EMF exposure condition. P-values less than 0.05 were considered as statistically significant.

### Cells exposure, exposure set-up, and dosimetry

Cells were exposed for 24 h in 96-well tissue culture plates (TCP) at SAR levels of 0.25, 1, and 4 W/kg. Intermittent exposure (5 min ON/10 min OFF) at the same average SAR level as with the continuous wave (CW) mode was implemented to mimic actual real-life exposure and help detect potential nonthermal bioeffects. RF EMF sham exposures were also performed under identical experimental conditions but with the generator turned off, i.e. at SAR equal to 0 W/kg. A novel exposure system, recently designed and characterized, was used for the first time for cells exposures to 5G-modulated 3.5 GHz signals^34^. The system was based on a cell culture incubator that allowed maintaining the desired biological conditions of 37 °C and 5% CO_2._ Comprehensive characterization of the system, through experimental measurements and numerical simulations has been described in detail elsewhere^34^. Briefly, a 150-l incubator (BINDER Gmbh, Tullingen, Germany), made of stainless-steel walls, was used as a reverberation chamber, i.e. a metallic large, closed cavity, with a high *Q*-factor, where a statistically homogeneous, randomly polarized, and isotropic field distribution was achieved via mechanical stirring of the field components^35^. Electromagnetic signal at 3.5 GHz was delivered to the biological samples through a printed patch antenna. A plastic holder with five levels was used to accommodate and simultaneously expose ten TCPs of 6- or 96- wells i.e. two per holder level. Each well of the 6- and 96- well TCPs was filled with 2 ml and 200 µl of cell culture medium, respectively. To ensure experimental reproducibility during exposure, the incubator was loaded with the same configuration used for the electromagnetic characterization, i.e. four and six 6 -and 96-well TCPs, respectively, due to the high SAR dependence on the chamber load.

The signal generation unit, composed of a RF signal generator (SMBV100A, Rohde & Schwarz, Munich, Germany), a 45-dB gain amplifier (Mini-circuits, ZHL-16W-43 + , NY, USA), a power circulator (Pasternack, PE83CR1005, CA, USA), and a bidirectional coupler (Mini-circuits, ZGBDC30-372HP + , NY, USA), was located outside the incubator. In addition, to ensure the continuous monitoring of the desired input power into the chamber, incident and reflected powers were monitored with a power meter (Agilent N1912A, USA) connected to the bidirectional coupler.

Local SAR was experimentally retrieved through temperature measurements of the RF-EMF induced heating recorded with a fiber-optic probe (Luxtron One, Lumasense Technologies, CA, USA). Measured SAR in the 96-well TCP was around 1 W/kg per watt antenna input power. To validate our systems, numerical simulations were performed using the finite difference time domain (FDTD)-based electromagnetic methodology^36^. The results of simulations were averaged over 50 positions of the stirrer, corresponding to a complete rotation. Although numerical simulations might not guarantee the absence of hotspots at specific locations of the exposed wells, the continuous stirring of the field components via the mechanical rotation of the metallic stirrer ensured the achievement of a good SAR homogeneity with variation within 30%. Overall, we showed that experimental and numerical SARs were in good agreement with differences < 30% considering the standard deviation that is compliant with ICNIRP guidelines^3^. According to measured and simulated values normalized to 1 W, incident power during biological exposure was adjusted to obtain required exposure levels of 0.25, 1, and 4 W/kg in a 96-well tissue culture plate. Measurements of the induced temperature elevation of the exposed medium were also performed using the Luxtron probe (Lumasense) under the specific cellular exposure condition of the study, showing a temperature increase of 1.7 °C at 4 W/kg, 0.7 °C at 1 W/kg and a negligeable temperature increase below 0.1 °C at 0.25W/kg using a continuous RF exposure, and a temperature increase of 0.8 °C at 4 W/kg, 0.3 °C at 1 W/kg and less than 0.1 °C at 0.25 W/kg using an intermittent (5 min ON, 10 min OFF) RF exposure. The temperature of the incubator was decreased accordingly to maintain the biological samples at 37 °C. Temperature stability of cell cultures at the bottom of the culture wells during the whole RF sessions at the various SAR levels was carefully assessed in a set of separate plates (See Fig. 1B,C for typical temperature traces obtained at 4 W/kg under continuous and intermittent exposure conditions, respectively). As expected, the temperature of the cell culture exposed to the intermittent signal is slightly waving (Fig. 1C). Of note, injection of the chemical triggered a transient drop in cell culture temperature by less than 0.5 °C as exemplified in Fig. 1B.

## Results

The hypothesis that 5G-modulated 3.5 GHz RF-EMF can impact HSF1, RAS, ERK, or PML basal or chemically-induced activity was tested using BRET-based assays previously described by our team^13,14,26^. BRET is a cell-based assay for studying protein–protein interactions and protein conformational changes in real-time and live cells. This technique relies on Forster resonance energy transfer from a bioluminescent donor to a fluorescent acceptor, both fused to the proteins of interest. In our experiment design, BRET probes were expressed in live cells. Since the fibroblast and keratinocyte cell lines used in this study are more difficult to transfect than the HEK293T cells used in our former studies, we systematically replaced the rLuc2 protein in our BRET assays with the brighter nanoLuciferase (nLuc)^28^. We also replaced the fluorescent acceptor sYFP2 with mNeonGreen (mNeonG) in all our assays because of the greater overlap between nLuc emission spectra and mNeonG excitation spectra^37^.

### Impact of 5G-modulated 3.5 GHz RF-EMF exposure on basal or chemically-induced HSF1 trimerization in XP6BE fibroblasts cells

**Figure 2:**
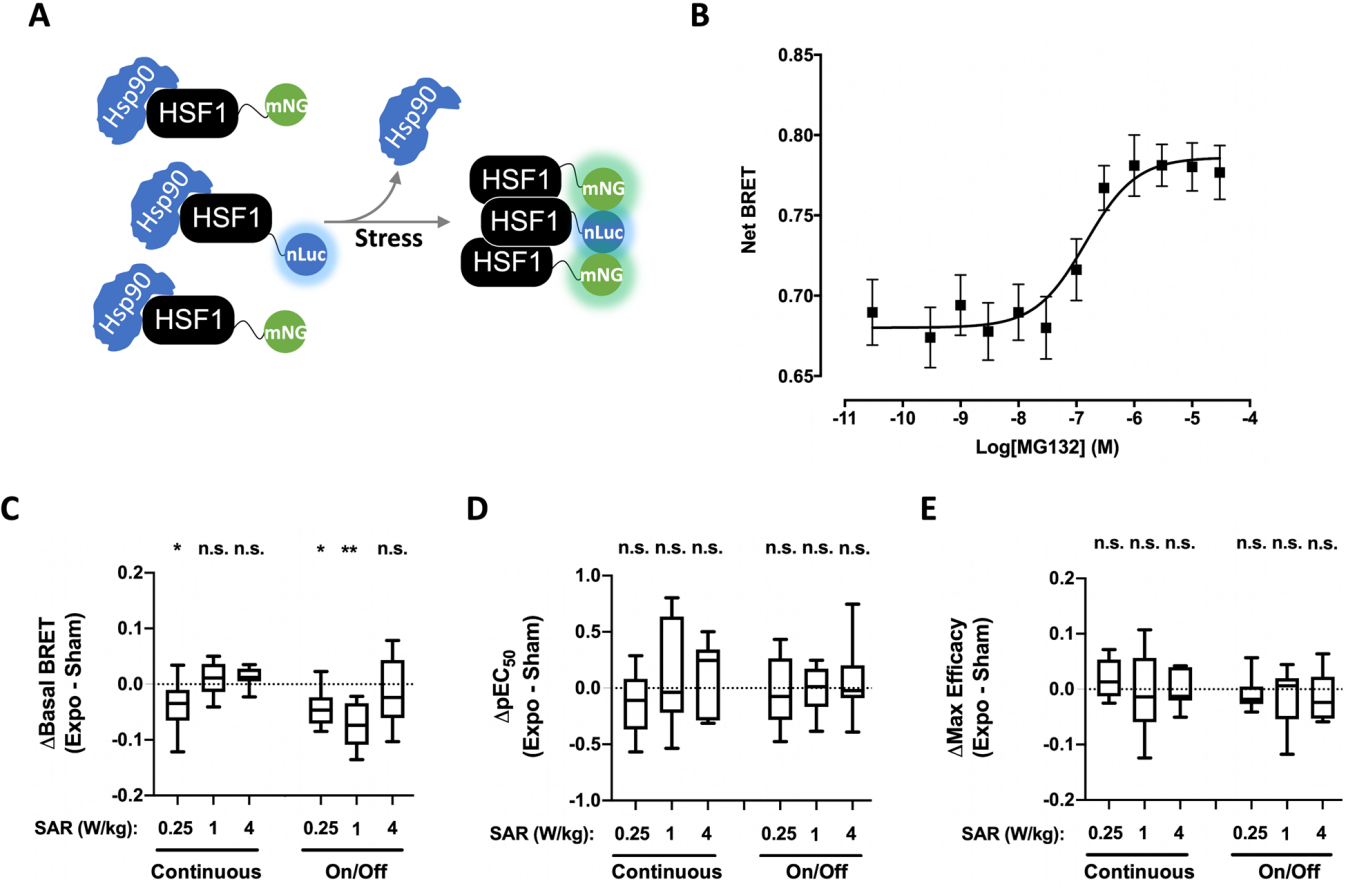
Effect of continuous or intermittent 5G signal exposure on HSF1 activation in XP6BE fibroblasts. (**A**) Mode of action of the intermolecular HSF1 BRET assay. (**B**) Dose–response curves of MG132-induced changes in nLuc-HSF1/mNeonG-HSF1 BRET signal. XP6BE fibroblasts cells transiently co-expressing nLuc-HSF1 and mNeonG-HSF1 proteins were activated for 18 h at 37 °C with increasing concentration of MG132 before BRET measurement. The results represent the average ± S.E.M. of 10 independent experiments done in duplicate. The pEC50 of MG132 was 6.83 ± 0.25 while the maximal efficacy of MG132 was 0.106 ± 0.018. (**C**–**E**) XP6BE skin fibroblasts transfected with the nLuc-HSF1/mNeonG-HSF1 BRET probe were sham-exposed or exposed to 5G-modulated 3.5 GHz at 0.25, 1 or 4 W/kg for 24 h either continuously or intermittently (5 min ON/10 min OFF). Cells were activated using increasing concentrations of MG132 under sham or RF-EMF exposure for the last 18 h of the RF-EMF exposure period before BRET measurement were performed. The results in panels C-E represent the Box and whisker plots of the basal BRET variation (**C**), the MG132-potency variation (**D**) and the MG132-maximal efficacy variation (**E**) between the 5G RF-EMF exposed- (Expo) and sham- conditions in both continuous and intermittent (on/off) exposure mode. Statistical significance of the derivation from the null hypothesis (no difference between sham and RF-EMF exposure) was assessed using the one-Sample Wilcoxon Signed Rank Test. n = 6–12 depending on the experimental condition. *n.s*. not significant; *p < 0.05; **p < 0.01.

To monitor HSF1 activity, we previously designed an intermolecular BRET test allowing the follow-up of HSF1 trimerization^13^, a key event on the roadmap of HSF1 activation in response to stress conditions such as heat-stress, oxidative-stress or proteotoxic-stress^38^. In this assay, N-terminally nLuc-tagged HSF1 is co-expressed with mNeonG-tagged HSF1 into a given cell line. Given that HSF1 trimerizes upon activation, the resulting BRET signal increases following HSF1 activation since trimerization brings donor and acceptor groups in close proximity^13^ (Fig. 2A).

To assess the functionality of this assay in skin fibroblasts, we co-expressed nLuc-HSF1 with mNeonG-HSF1 in the XP6BE fibroblast cell line^39^, and challenged the transfected cells with increasing concentrations of the proteasome inhibitor MG132 to trigger a proteotoxic stress^40^. As expected, MG132 induced a concentration-dependent increase in the basal BRET signal with an EC_50_ in the hundred nanomolar range (Fig. 2B), showing the assay's effectiveness.

We then assessed whether continuous or intermittent (5 min ON/10 min OFF) cell exposure to 5G-modulated 3.5 GHz RF-EMF signal at 0.25, 1, and 4 W/kg under isothermal conditions for 24 h impacted the nLuc-HSF1/mNeonG-HSF1 basal BRET signal, or either the MG132 potency or efficacy to activate HSF1 in the transfected XP6BE fibroblast cell line. Interestingly, we measured few but significant and reproducible decrease of the HSF1 basal BRET when the XP6BE fibroblast cell line was continuously exposed at 0.25 W/kg for 24 h or intermittently exposed at 0.25 and 1 W/kg for 24 h (Fig. 2C). While this BRET signal decrease represents only ~ 6 to 10% of the basal BRET measured, it proportionally corresponds to ~ 37 to 61% (in absolute terms) of the effect triggered by MG132 (Tables 1 and 2). No effect was measured at 4 W/kg whether the signal was emitted continuously or intermittently. Also, exposure to the RF-EMF signal changed neither the MG132 potency nor its efficacy to activate HSF1, whatever the SAR and the continuous or intermittent emission mode of the signal (Fig. 2D,E, Tables 3 and 4).

**Table 1:** Percentage of RF EMF-induced basal BRET change relative to the basal BRET under the sham condition.

	SAR (W/kg)	HSF1	RAS	ERK	PML
Continuous	0.25	− 5.4%* (12)	ns (12)	ns (12)	ns (12)
	1	ns (8)	ns (7)	ns (6)	ns (8)
	4	ns (7)	ns (6)	ns (7)	− 3.1* (7)
Intermittent	0.25	− 5.9%* (8)	ns (8)	ns (8)	ns (8)
	1	− 9.5%** (8)	ns (9)	ns (9)	ns (9)
	4	ns (13)	ns (13)	ns (13)	ns (13)

Data were acquired in XP6BE skin fibroblasts transfected with the indicated BRET probe and either sham-exposed or exposed for 24 h to continuous or intermittent 5G-modulated 3.5 GHz RF-EMF at 0.25, 1 or 4 W/kg. The number of independent experiments performed is indicated in brackets. *ns* no significant. *p < 0.05. **p < 0.01.

**Table 2:** Percentage of RF EMF-induced basal BRET change relative to the maximal efficacy of MG132, PMA and As_2_O_3_ to activate HSF1, RAS/ERK and PML, respectively.

	SAR (W/kg)	HSF1	RAS	ERK	PML
Continuous	0.25	− 34.9%* (12)	ns (12)	ns (12)	ns (12)
	1	ns (8)	ns (7)	ns (6)	ns (8)
	4	ns (7)	ns (6)	ns (7)	− 14.9%* (7)
Intermittent	0.25	− 37.7%* (8)	ns (8)	ns (8)	ns (8)
	1	− 61.3%** (8)	ns (9)	ns (9)	ns (9)
	4	ns (13)	ns (13)	ns (13)	ns (13)

Data were acquired in XP6BE skin fibroblasts transfected with the indicated BRET probe and exposed for 24 h to continuous or intermittent 5G-modulated 3.5 GHz RF-EMF at 0.25, 1 or 4 W/kg. The number of independent experiments performed is indicated in brackets. *ns* no significant. *p < 0.05. **p < 0.01.

**Table 3:** RF EMF-induced change in MG132, PMA and As_2_O_3_ pEC_50_ values measured in XP6BE skin fibroblasts transfected with the indicated BRET probe following continuous or intermittent exposure to 5G-modulated 3.5 GHz RF-EMF at 0.25, 1 or 4 W/kg for 24 h (for each probe, the corresponding drug used in dose–response experiments in indicated).

	SAR (W/kg)	HSF1 (MG132)	RAS (PMA)	ERK (PMA)	PML (As_2_O_3_)
Continuous	0.25	ns (12)	ns (12)	− 0.27* (12)	ns (12)
	1	ns (8)	ns (7)	ns (6)	ns (8)
	4	ns (7)	ns (6)	ns (7)	ns (7)
Intermittent	0.25	ns (8)	ns (8)	ns (8)	ns (8)
	1	ns (8)	ns (9)	ns (9)	ns (9)
	4	ns (13)	ns (13)	ns (13)	ns (13)

The number of independent experiments performed is indicated in brackets. *ns* no significant. *p < 0.05.

**Table 4:** Percentage of RF EMF-induced change in the MG132, PMA and As_2_O_3_ maximal efficacy to activate HSF1, RAS/ERK and PML, respectively.

	SAR (W/kg)	HSF1 (MG132)	RAS (PMA)	ERK (PMA)	PML (As_2_O_3_)
Continuous	0.25	ns (12)	ns (12)	ns (12)	− 17.4%* (12)
	1	ns (8)	ns (7)	ns (6)	− 19.9%* (8)
	4	ns (7)	ns (6)	ns (7)	− 23.6%* (7)
Intermittent	0.25	ns (8)	ns (8)	ns (8)	ns (8)
	1	ns (8)	ns (9)	ns (9)	ns (9)
	4	ns (13)	ns (13)	ns (13)	ns (13)

Data were acquired in XP6BE skin fibroblasts transfected with the indicated BRET probe and exposed for 24 h to continuous or intermittent 5G-modulated 3.5 GHz RF-EMF at 0.25, 1 or 4 W/kg. *ns* no significant changes of the basal BRET has been detected. *p < 0.05. The number of independent experiments performed is indicated in parenthesis.

### Impact of 5G-modulated 3.5 GHz RF-EMF exposure on basal or chemically-induced RAS and ERK activities in XP6BE fibroblasts cells

**Figure 3:**
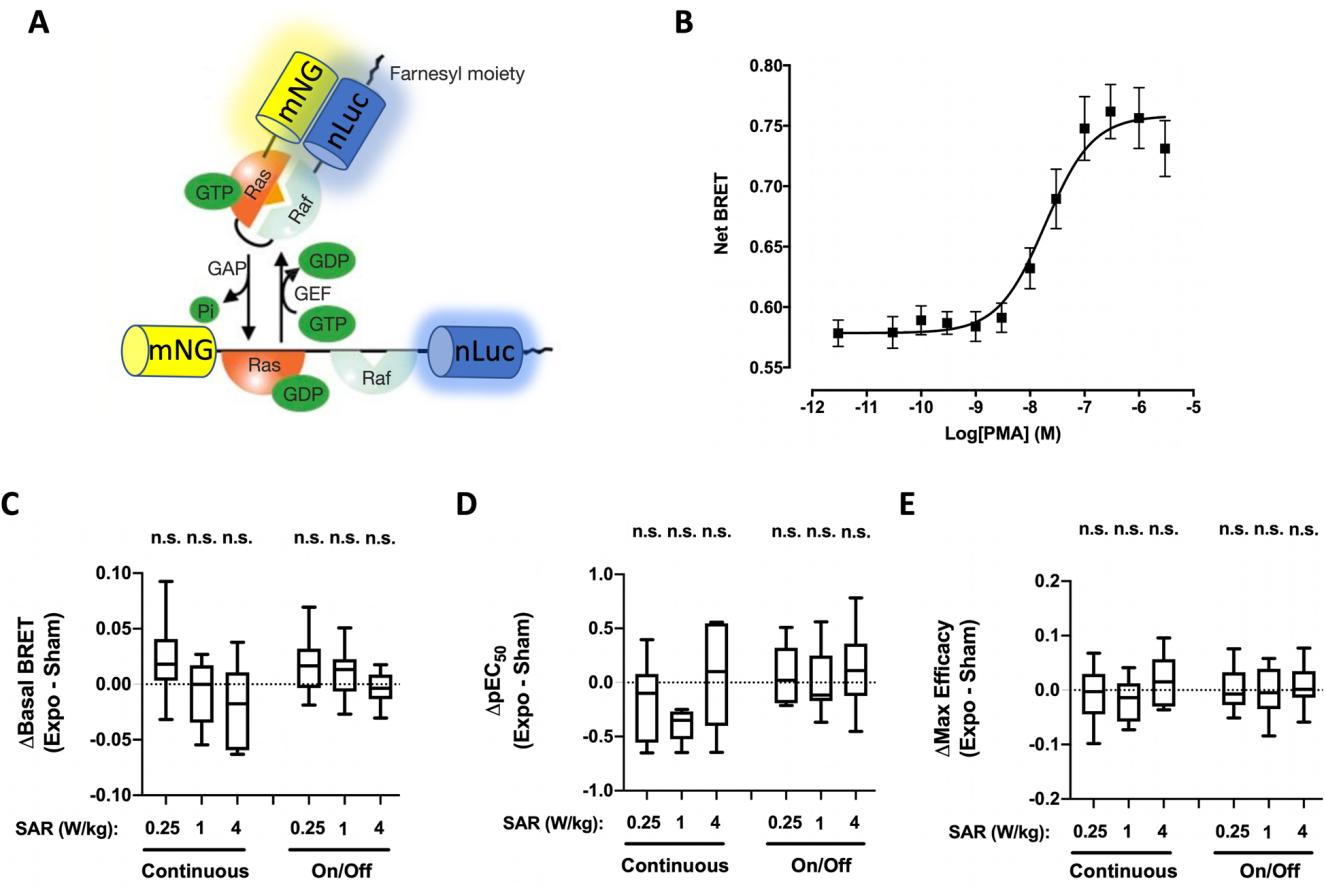
Effect of continuous or intermittent 5G RF-EMF exposure on RAS activation in XP6BE skin fibroblasts. (**A**) Mode of action of the RAS BRET biosensor. (**B**) Dose–response curves of PMA-induced change in RAS activity using the pRaichuEV-Ras BRET probe. XP6BE fibroblasts were activated for 15 min at 37 °C with increasing concentration of PMA before BRET measurement. The results represent the average ± SEM of 10 independent experiments done in duplicate. The pEC50 of PMA was 7.60 ± 0.17 while the maximal efficacy of PMA was 0.107 ± 0.012. (**C**–E) XP6BE fibroblasts cells transfected with the pRaichuEV-Ras BRET probe were sham-exposed or exposed to 5G-modulated 3.5 GHz at 0.25, 1 or 4 W/kg for 24 h, either continuously or intermittently (5 min ON/10 min OFF). Cells were activated using increasing concentrations of PMA under sham or RF-EMF exposure for the last 15 m before BRET measurement were performed. The results in panels C-E represent the Box and whisker plots of the basal BRET variation (**C**), the PMA-potency variation (**D**) and the PMA-maximal efficacy variation (**E**) between the 5G RF-EMF exposed- (Expo) and sham- conditions in both continuous or intermittent exposure mode. Statistical significance of the derivation from the null hypothesis (no difference between sham and RF-EMF exposure) was assessed using the one-Sample Wilcoxon Signed Rank Test. n = 6–12 depending on the experimental condition. *n.s.* not significant; *p < 0.05; **p < 0.01.

To assess the potential impact of 5G-modulated 3.5 GHz RF-EMF exposures on basal- or chemically-induced RAS activity, XP6BE fibroblast cells were transfected with a BRET probe consisting in sandwiching the H-Ras and the Ras-Binding Domain of Raf (Raf RBD) in-between nLuc and mNeonGreen. This molecular BRET probe is derived from the Fluorescence Resonance Energy Transfer (FRET)- probe described by the Matsuda laboratory^27,41^, and relies on the rapprochement of nLuc and mNeonG following the binding of GTP-Ras to Raf RBD (Fig. 3A).

**Figure 4:**
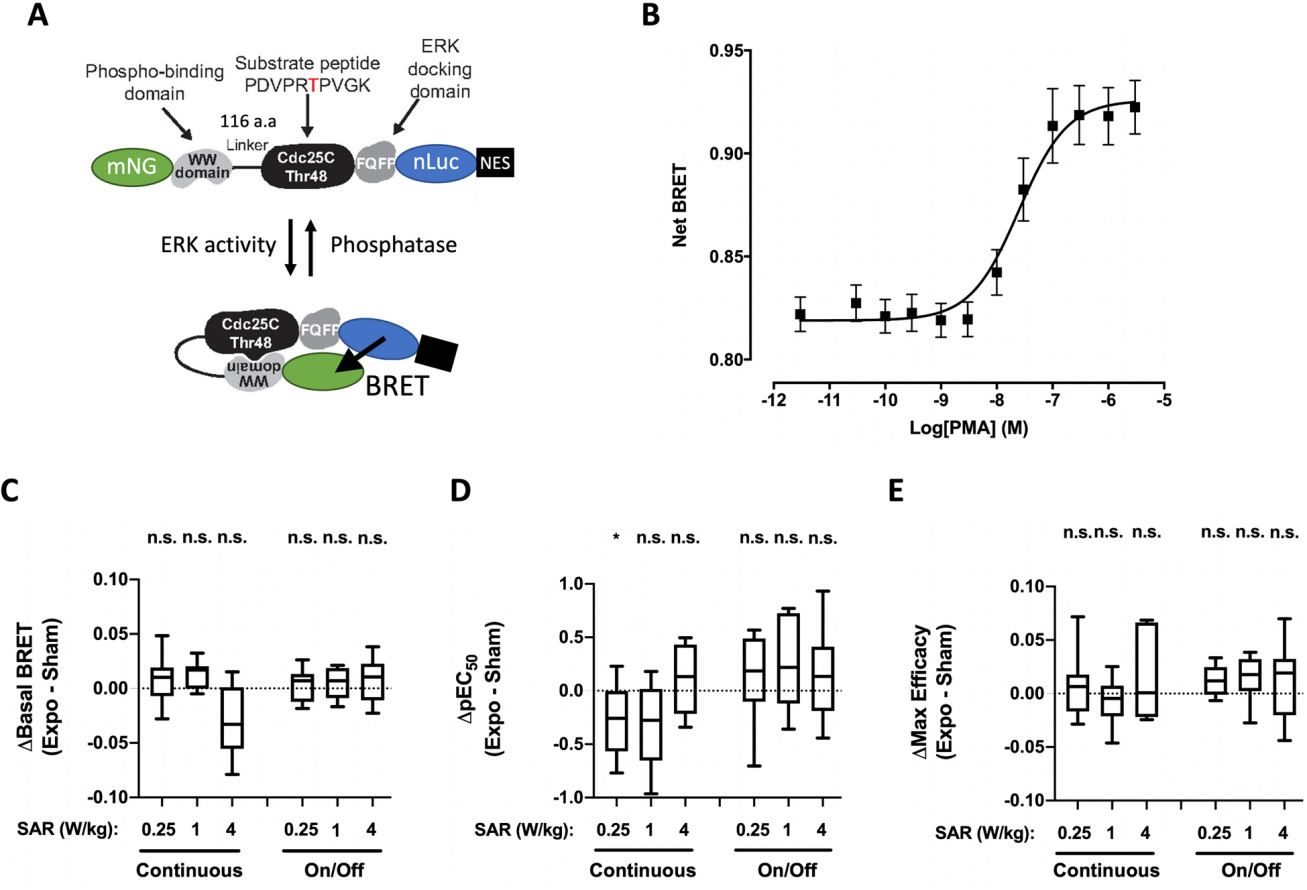
Effect of continuous or intermittent 5G RF-EMF exposure on ERK activation in XP6BE skin fibroblasts. (**A**) Mode of action of the ERK BRET biosensor. (**B**) Dose–response curves of PMA-induced change in ERK activity using the pEKAREV BRET probe. XP6BE fibroblasts were activated for 15 min at 37 °C with increasing concentration of PMA before BRET measurement. The results represent the average ± SEM of 10 independent experiments done in duplicate. The pEC50 of PMA was 7.72 ± 0.15 while the maximal efficacy of PMA was 0.178 ± 0.018. (**C**–**E**) XP6BE fibroblast cells transfected with the pEKAREV BRET probe were sham-exposed or exposed to 5G-modulated 3.5 GHz at 0.25, 1 or 4 W/kg for 24 h, either continuously or intermittently (5 min ON/10 min OFF). Cells were activated using increasing concentrations of PMA under sham or RF-EMF exposure for the last 15 m before BRET measurement were performed. The results in panels C-E represent the Box and whisker plots of the basal BRET variation (**C**), the PMA-potency variation (**D**) and the PMA-maximal efficacy variation (**E**) between the 5G RF-EMF exposed- (Expo) and sham- conditions in both continuous or intermittent exposure mode. Statistical significance of the derivation from the null hypothesis (no difference between sham and RF-EMF exposure) was assessed using the one-Sample Wilcoxon Signed Rank Test. n = 6–12 depending on the experimental condition. *n.s*. not significant; *p < 0.05; **p < 0.01.

Similarly, the potential impact of 5G-modulated 3.5 GHz RF-EMF signal exposure on basal- or chemically-induced ERK activity was assessed in XP6BE fibroblast cells transfected with a BRET probe comprising a ERK sensor domain and a ERK ligand domain connected by a flexible linker, but sandwiched by a mNeonG and nLuc instead of two fluorescent energy acceptor and donor as initially described^27^ (Fig. 4A). Once activated, endogenous ERK proteins phosphorylate the sensor part of this BRET probe. This triggers the sensor domain interaction with the ligand domain, thereby inducing the biosensor closure onto itself. Such conformational change brings nLuc in close proximity to mNeonGreen, thereby increasing the BRET efficiency.

XP6BE fibroblasts transiently expressing these RAS and ERK-sensitive BRET probes were therefore challenged for 15 min with phorbol-12-myristate-13-acetate (PMA), a well-known phorbol-ester mimicking diacylglycerol leading to PKC-dependent activation of RAS and ERK^42^. As expected, PMA induced a concentration-dependent increase of the BRET measured with the RAS (Fig. 3B) and ERK (Fig. 4B) BRET probes. Furthermore, for both BRET probes, the measured EC_50_ were in the tens of nanomolar range, again demonstrating the high sensitivity of our BRET-based assays to monitor RAS and ERK activation.

Continuous or intermittent exposure of XP6BE fibroblasts transiently expressing the RAS and ERK probes to 5G-modulated 3.5 GHz RF-EMF signal for 24 h neither impacted RAS and ERK basal BRET (Figs. 3C and 4C, Table 1 and 2) nor PMA potency (Figs. 3D and 4D, Table 3) or maximal efficacy (Figs. 3E or 4E, Table 4) to activate these kinases. We only measured a slight decrease in the PMA potency to activate ERK in XP6BE fibroblasts continuously exposed for 24 h at a SAR of 0.25 W/kg (Fig. 4D, Table 3).

### Impact of 5G-modulated 3.5 GHz RF-EMF exposure on basal or chemically-induced PML SUMOylation in XP6BE fibroblasts cells

**Figure 5:**
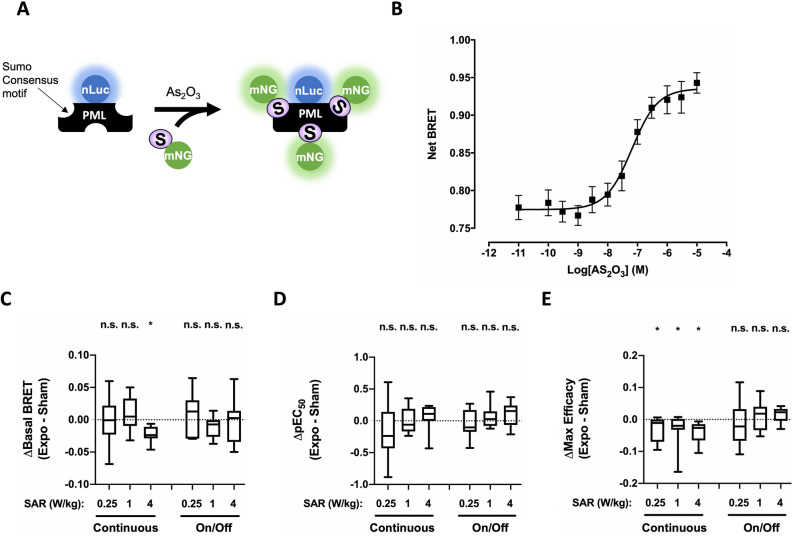
Effect of continuous or intermittent 5G-EMF exposure on PML SUMOylation in XP6BE skin fibroblasts. (**A**) Schematic description of the intermolecular BRET assays for the detection of PML SUMOylation. (**B**) Dose–response curves of As_2_O_3_-induced PML SUMOylation using the PML-nLuc/mNeonGreen-SUMO1 intermolecular assay. XP6BE fibroblasts were activated for 4 h at 37 °C with increasing concentration of As_2_O_3_ before BRET measurement. The results represent the average ± SEM of 10 independent experiments done in duplicate. The pEC_50_ of As_2_O_3_ was 7.19 ± 0.15 while the maximal efficacy of PMA was 0.161 ± 0.014. (**C**–**E**) XP6BE fibroblasts co-transfected with the mammalian expression vector encoding PML-nLuc and mNeonGreen-SUMO1 were sham-exposed or exposed to 5G modulated 3.5 GHz at 0.25, 1 or 4 W/kg for 24 h, either continuously or intermittently (5 min ON/10 min OFF). Cells were activated using increasing concentrations of As_2_O_3_ under sham or RF-EMF exposure for the last 4 h before BRET measurement were performed. The results in panels C-E represent the Box and whisker plots of the basal BRET variation (**C**), the As_2_O_3_-potency variation (**D**) and the As_2_O_3_-maximal efficacy variation (**E**) between the 5G RF-EMF exposed- (Expo) and sham- conditions in both continuous or intermittent exposure mode. Statistical significance of the derivation from the null hypothesis (no difference between sham and RF-EMF exposure) was assessed using the one-Sample Wilcoxon Signed Rank Test. n = 6–12 depending on the experimental condition. *n.s*. not significant; *p < 0.05; **p < 0.01.

Finally, knowing that post-translational covalent addition of SUMO to PML, a process known as SUMOylation, is a key-event leading to PML activation and the formation of PML NBs, we used an intermolecular BRET assay^26^ to assess whether continuous or intermittent 5G-modulated 3.5 GHz RF-EMF signal exposure may affect PML activity. In this assay, we measured the BRET signal between a SUMO1 protein N-terminally tagged with mNeonGreen and a PMLIII protein C-terminally tagged to nLuc (Fig. 5A). XP6BE fibroblasts transiently transfected with PMLIII-nLuc/mNeonG-SUMO1 expression vectors were therefore challenged with arsenic trioxide (As_2_O_3_)_,_ a well-known oxidative-stress inducer in cells^43^ that efficiently triggers PML SUMOylation^26,44^. As expected, As_2_O_3_ dose-dependently increased the BRET signal between PMLIII-nLuc and mNeonG-SUMO1 in XP6BE fibroblasts cells with an EC_50_ in the tens of nanomolar range (Fig. 5B), indicating an efficient PML SUMOylation in these cells. Furthermore, neither basal PML SUMOylation nor As_2_O_3_ potency or maximal efficacy to trigger PML SUMOylation was affected following intermittent exposure of transiently transfected XP6BE fibroblasts to 5G-modulated 3.5 GHz RF-EMF signal for 24 h, whatever the SAR tested (Fig. 5C–E). We, however, measured a slight but reproductive decrease in the basal PML SUMOylation when XP6BE fibroblasts were continuously exposed for 24 h at 4 W/kg (Fig. 5C). This variation stood for less than 3.5% of the PML basal BRET signal (Table 1) and for 14.9% of the As_2_O_3_ maximal efficacy (in absolute terms) (Table 2). Also, continuous exposure for 24 h did not change As_2_O_3_ potency to trigger PML SUMOylation (Fig. 5D, Table 3), but led to a slight decrease in As_2_O_3_ maximal efficacy when compared to the condition (Fig. 5E, Table 4).

### Checking the absence of BRET signals variation following the interruption of exposure to RF-EMF

**Figure 6:**
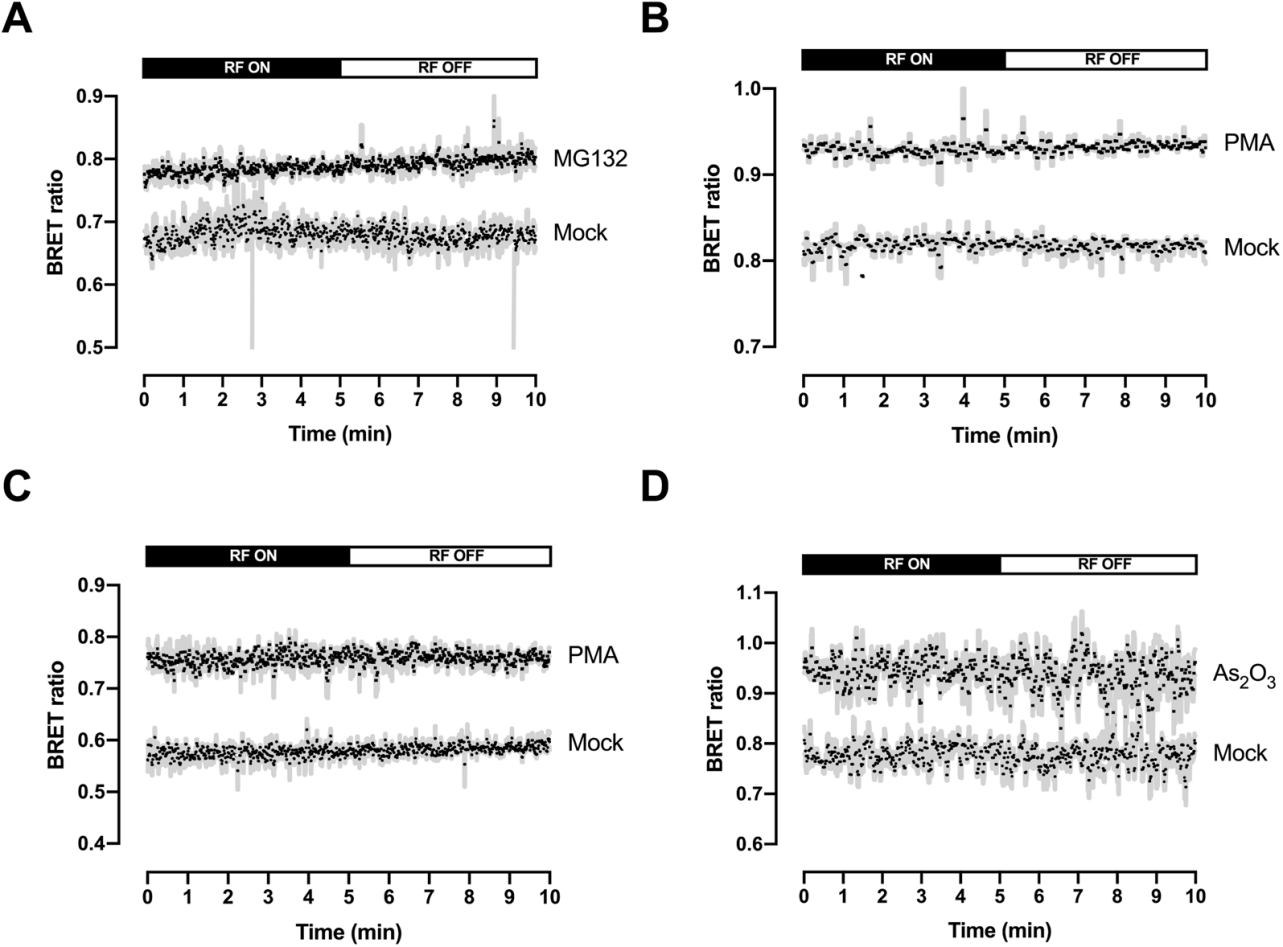
Real-time monitoring of the BRET variation after RF-EMF shutdown. HEK293T cells that were transfected with either the HSF1 (**A**), RAS (**B**), ERK (**C**) or PML (**D**) BRET probes were exposed to a 5G-modulated 3.5 GHz at 4 W/kg for 24 h and either mock-treated or treated with 1 µM of PMA, 10 µM of As_2_O_3_ or 10 µM of MG132 according to the timeline given in Fig. 1A. Coelenterazine H was injected into the cell culture media 10 min before the end of the RF-EMF exposure. BRET signals were remotely measured for the last 5 min before the end of RF-EMF exposure and during the next 5 min after the end of RF-EMF exposure. For each BRET probe, the kinetic of the BRET signal evolution is shown in mock-treated and drug-treated cell, and represents the average of 3–4 independent experiments.

We next assessed wether we had not missed a potential effect of RF exposure that might have disappeared during the short time laps between the end of the exposure and the completion of the BRET reading (less than 5 min). To verify this hypothesis, coelenterazine H was added to the cell culture wells to start the bioluminescent reaction 10 min before the end of cells exposure to 5G-modulated 3.5 GHz signal emitted continuously at 4 W/kg. Then, using an optical fiber, we remotely measured the BRET ratio in real-time during the 5 last min of the 24 h RF-EMF exposure period and kept measuring during the 5 next min without RF exposure. As indicated in Fig. 6, whatever BRET probe considered, and in the presence or absence of chemical activation, we did not detect any variation of the BRET signal after the end of RF exposure, thereby validating the results previously obtained.

### Impact of 5G-modulated 3.5 GHz RF-EMF exposure on basal or chemically-induced HSF1, RAS/ERK and PML activities in HaCAT keratinocyte cells

**Figure 7:**
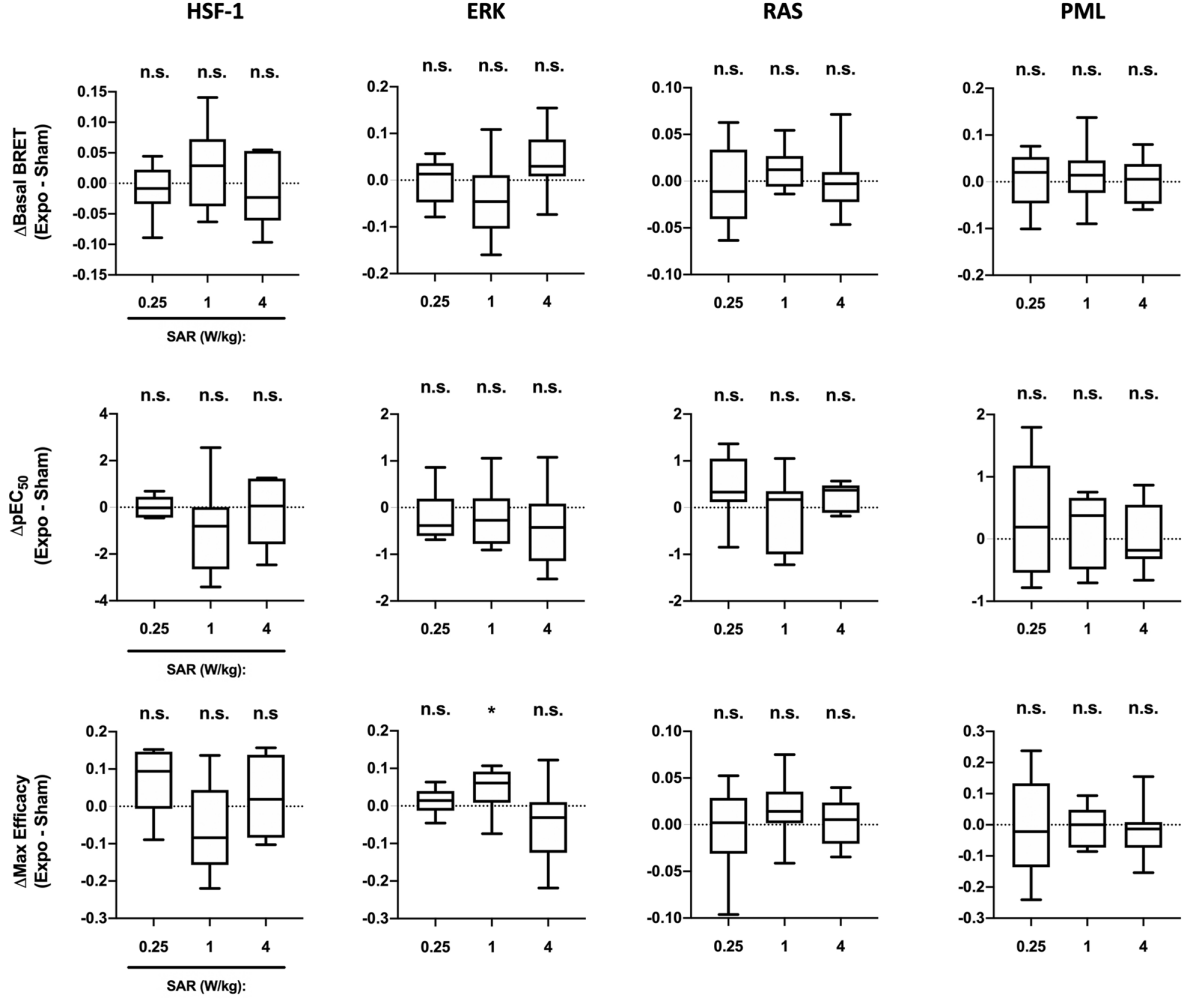
Effect of continuous or intermittent 5G RF-EMF exposure on HSF1, ERK, RAS and PML activities in HaCaT keratinocytes. HaCaT keratinocytes cells transiently expressing HSF1, ERK, RAS and PML constructs were sham-exposed or exposed to 5G-modulated 3.5 GHz at 0.25, 1 or 4 W/kg for 24 h, either continuously or intermittently (5 min ON/10 min OFF). Cells were activated using increasing concentrations of either MG132 (HSF1), PMA (RAS and ERK) or As_2_O_3_ (PML) under sham or RF-EMF exposure for the last 18 h (HSF1), 15 min (RAS and ERK) or 4 h (PML) of the RF-EMF exposure period. RF-EMF exposure was then shut-down and BRET signals were measured immediately. For each dose response curve, basal BRET signal, potency and maximal efficacy of the activating chemical agent were derived and reported as boxes and whisker plots of the variation between the 5G RF-EMF exposed- (Expo) and sham- conditions in both continuous or intermittent exposure mode. Statistical significance of the derivation from the null hypothesis (no difference between sham and RF-EMF exposure) was assessed using the one-Sample Wilcoxon Signed Rank Test. n = 6–13 depending on the experimental condition. *n.s*. not significant; *p < 0.05; **p < 0.01.

In a further effort to study the potential effect of 5G signal on skin cells and given that the upper layer of the skin (epidermis) is primarily composed of keratinocytes, we performed a new set of experiments using the HaCaT keratinocyte cell line to assess whether a 24 h continuous exposure to 5G-modulated 3.5 GHz RF-EMF may impact HSF1, RAS, ERK, and PML activities. HaCaT cells transiently transfected with the BRET probes probing HSF1, RAS, ERK, and PMLIII were respectively challenged with increasing concentration of MG132 (for HSF1), PMA (for RAS and ERK), and As_2_O_3_ (for PML) following sham- or continuous-exposure to 5G-modulated 3.5 GHz RF-EMF for 24 h at three different SAR of 0.25, 1, and 4 W/kg. Dose–response curves were generated for each experimental condition (See Supp. Fig. 2 for the curves derived from the sham-exposed condition). Basal BRET of each probe and the potency and maximal efficacy of each chemical agent were therefore calculated, and the variation of each parameter between sham-exposed and RF-EMF exposed conditions was reported in Fig. 7. We found no difference between sham and exposed conditions, whatever the molecular target, the SAR, or the metric considered. The only exception was a slight increase in the maximal PMA efficacy to activate ERK when HaCaT cells were exposed at 1 W/kg.

## Discussion

In this study, we investigated whether continuous or intermittent (5 min ON / 10 min OFF) exposure to 5G-modulated 3.5 GHz RF-EMF signal at 0.25, 1, and 4 W/kg for 24 h under isothermal conditions impacted human skin fibroblast and keratinocyte cell stress response at the molecular level. Using our own existing BRET-based molecular probes, we focused on HSF1, RAS/ERK, and PML proteins which are at the crossroad of various molecular pathways, ensuring proper cell response to a wide array of environmental stress, including thermal injury, oxidative stress, and proteotoxic stress^38^. We assessed whether 5G-signal exposure affected either basal or chemically-activated activity for each of these cellular stress molecular markers.

We found that the HSF1 basal BRET level was reduced when XP6BE fibroblasts cells were respectively exposed to continuous and intermittent 5G signal for 24 h at 0.25 W/kg and to intermittent 5G signal for 24 h at 1 W/kg. No change in HSF1 basal BRET signal was detected at 1 W/kg when a continuous signal was used or when cells were continuously or intermittently exposed at 4 W/kg.

At first glance, the RF-EMF induced decrease of the HSF1 basal BRET signal may appear relatively small since it represents only 6–11% of the HSF1 basal BRET signal in the sham experiment (Fig. 2B). However, since chemical activation with MG132 only increased the basal BRET by 0.1 BRET unit (i.e. a 14% increase of the basal BRET) (Fig. 2B), the effect of 5G RF-EMF exposure appear important in term of absolute values. Interestingly, these results parallel the ones previously obtained by us with HEK293T cells exposed for 24 h to either unmodulated (continuous wave, CW) or GSM-modulated 1.8 GHz signal^13^. In this former work, RF-EMF exposure slightly decreased the HSF1 basal activity at the lower SAR tested (1.5 W/kg) but not at higher SAR (4 W/kg). Whether the effect of RF-EMF on basal HSF1 may impact the physiological HSF1-dependent stress response in RF-EMF exposed organisms remains to be determined. In this previous in-vitro study, while we detected that MG132 maximal efficacy to trigger HSF1 trimerization in HEK293T cells was increased following 24 h exposure to CW or GSM signals at 1.5 W/kg and to CW signal at 6 W/kg^13^, we did not detect any variation of the MG132 potency or efficacy to trigger HSF1 activation following 5G RF-EMF exposure (Fig. 2).

Considering the other BRET assays performed on skin fibroblasts, we only detected a slight leftward shift, less than a quarter of a log, of PMA potency to activate ERK at 0.25 W/kg and a small reduction in As_2_O_3_ efficacy to trigger PML SUMOylation, but only when cells were continuously exposed. No other RF-induced effect could be detected on 5G RF-EMF exposed fibroblasts, whatever the molecular probe considered and whatever the SAR or the signal envelope used. Finally, all over the assays performed on keratinocytes, we only detected a ~ 37% increase in PMA efficacy to activate ERK at 1 W/kg but not at 0.25 or 4 W/kg. Importantly, no changes in BRET measurement were detected following the end of RF-EMF exposure when the BRET signal was read in real-time in fibroblasts cells that were continuously exposed for 24 h to the 5G modulated 3.5 GHz at 4 W/kg, ruling-out a potential bias due to the time needed to read our BRET signal in 96-well plates after the end of RF-EMF exposure.

Altogether, these results can appear puzzling at several levels. Considering the cell types used, it is remarkable that RF-EMF exposure with two different carrier waves (1.8 and 3.5 GHz) and with different signal modulation (CW or GSM-modulation in our previous study and 5G-modulation in the present study) can consistently decrease HSF1 basal activity in HEK293T embryonic kidney cells^13^ and XP6BE skin fibroblasts (Fig. 2) but not in HaCaT keratinocytes (Fig. 7). Similarly, we detected that PMA maximal efficacy to activate ERK (i) decreased when HEK293T cells were exposed to a CW or a GSM-modulated 1.8 GHz signal, (ii) increased in keratinocytes, but (iii) was not impacted in 5G RF-EMF exposed skin fibroblasts. Finally, As_2_O_3_ maximal efficacy to trigger PML SUMOylation was slightly decreased in skin fibroblasts when exposed continuously but was not modified in keratinocytes.

Several authors have already reported qualitative or quantitative variations in some biomolecular effects induced in different cell lines following their exposure to RF-EMF signals in the GHz range under identical experimental conditions^45–47^. While it is fully expected that different cell types can respond differently to the same stimulus, to our knowledge, no research team has yet elucidated why and how low-level RF-EMF may impact living matter. Beside the lack of non-thermal mechanisms, it is also intriguing that the few RF-EMF induced effects herein detected on HSF1, ERK, and PML activities do not follow a classical dose–response profile. For example, the RF-EMF induced inhibition of HSF basal activity in human skin fibroblasts could only be detected at 0.25 W/kg but not at 4 W/kg, under both intermittent and continuous exposures. Strikingly, at 1 W/kg, there was no change in the HSF1 basal BRET when the cells were continuously exposed, while the HSF1 basal BRET was further decreased when the cells were intermittently exposed. Similarly, PMA maximal efficacy to activate ERK in keratinocytes was increased at 1 W/kg but neither at 0.25 W/kg nor 4 W/kg (Fig. 5). Only As_2_O_3_ maximal efficacy to trigger PML SUMOylation seemed to decrease dose-dependently with the SAR under continuous exposure, but the magnitude of this effect was small (Table 4).

May RF-EMF exposure at low levels elicit a non-thermal biological effect while RF-EMF exposure at higher levels may not? Several authors in this research field have already reported on the so-called "window" effect, where EMF exposure at specific intensities produces a given biological effect that could not be detected using EMF exposure to lower or higher intensities^48,49^. Unfortunately, no further experimental proofs were later published concerning such window effect and, accordingly, no explanation concerning the underlying molecular mechanism was provided by the authors.

More recently, Pooam et al. invoked a hormetic dose–response effect to explain that ROS production in HEK293T cells exposed to 1.8 GHz RF-EMF is maximal at an intermediate signal amplitude^50^. Hormesis is a toxicological concept characterized by a stimulated biological response when exposed to a low, subtoxic amount of stressor and by detrimental effects of high, toxic levels of the same stressor^51^. Furthermore, an impressive array of cytoprotective molecular mechanisms and signal transduction pathways have been shown to respond in a hormetic way, including the activation of HSF1 and ERK^52,53^. Therefore, whether RF-EMF exposure, alone or in combination with a chemical activating agent, could trigger or magnify a hormetic response on HSF1 or ERK activity needs to be studied more carefully. Interestingly, hormesis is also a time-dependent process^54^. Of note, a time-dependent hormetic effect has been proposed to explain that short exposure (1 h) to 1.8 GHz RF-EMF at an average SAR of 4.0 W/kg induces DNA fragmentation in mouse embryonic fibroblasts while more prolonged exposure (36 h) decreased DNA fragmentation to a lower level than for the sham condition^55^. Accordingly, since we only tested one exposure time (24 h), it will be interesting to repeat these experiments to assess potential time-dependent variation of the herein detected effects.

In conclusion, our BRET study shows no conclusive evidence that molecular effects can arise when skin cells are exposed to a 5G RF-EMF signal (3.5 GHz) for 24 h, even at levels above the ICNIRP guidelines for far-fields public exposure (0.08 W/Kg). Only a few statistically-significant changes were detected that depend on (i) the molecular probe used, (ii) the type of cells used, (iii) the presence of an activating chemical agent, with a most than probable time- and dose-dependency, and (iv) the characteristics of the RF-EMF exposure such as SAR, frequency, or modulation of the carrier wave. Given that we used more than 100 different experimental parameters and that all but one of the detected effects were within a risk of 5% error, it was statistically expected to have some false positive data. We reached a similar conclusion when we studied the effect of various 1.8 GHz signals on primary brain cell cultures and neuroblastoma cell lines using label-free techniques^56^. Therefore, apart from the effect of RF-EMF on HSF1 basal activity that may deserve further investigations, we found no sufficient evidence toward physiological effect of the tested RF-EMF alone or in combination with chemicals.

## Supplementary Information

Supplementary Figure 1.

Supplementary Figure 2.

## Data Availability

The datasets used and/or analyzed during the current study are available from the corresponding author on reasonable request.
