# Supplementary figures and images for: Effects of 5G-modulated 3.5 GHz radiofrequency field exposures on HSF1, RAS, ERK, and PML activation in live fibroblasts and keratinocytes cells
Source: Sci Rep. 2023 May 23;13:8305. doi: 10.1038/s41598-023-35397-w (PMC10203668; doi:10.1038/s41598-023-35397-w)

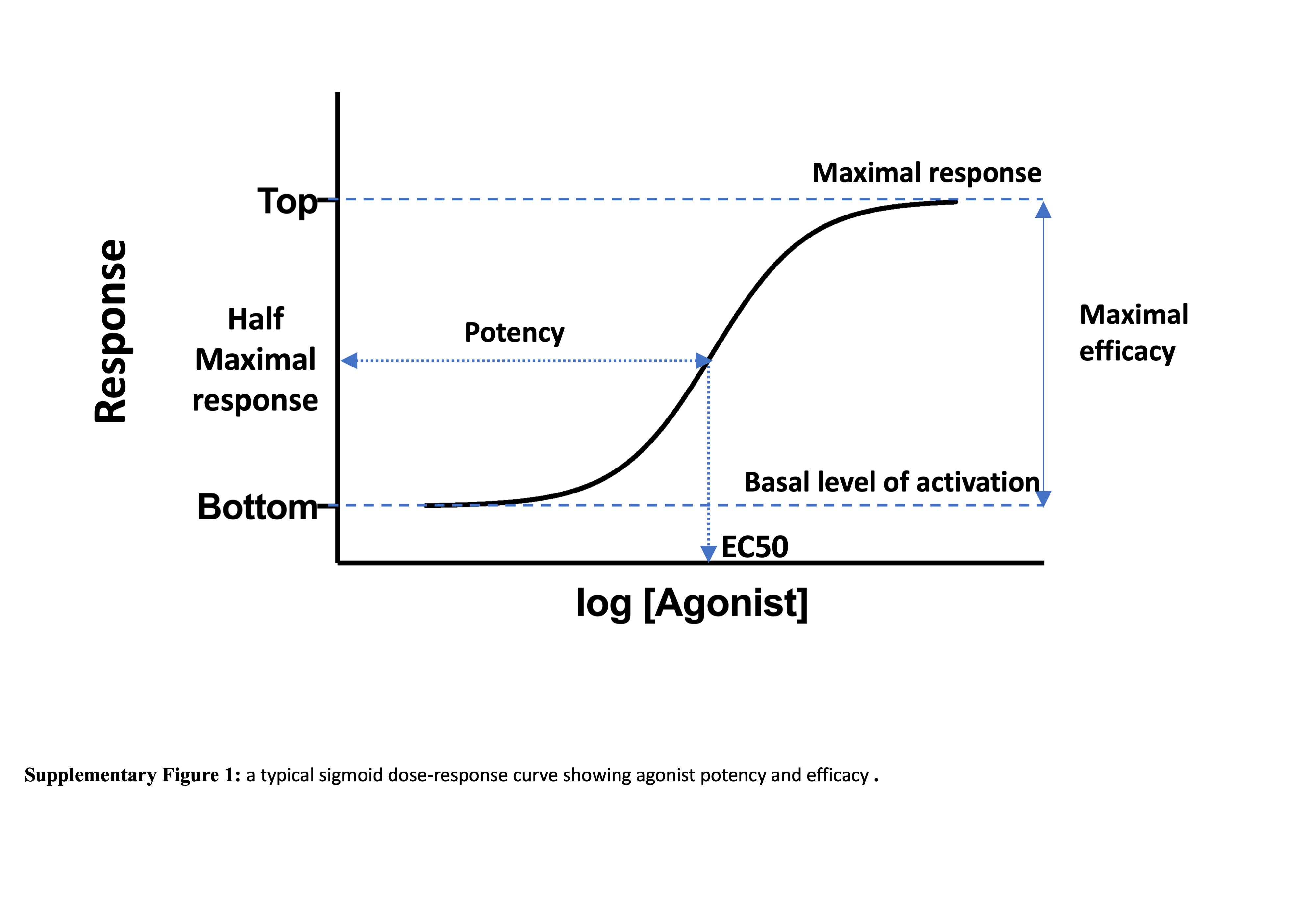

Supplement: Supplementary file 1 — Supplementary Figure 1. [file 41598_2023_35397_MOESM1_ESM.tiff]

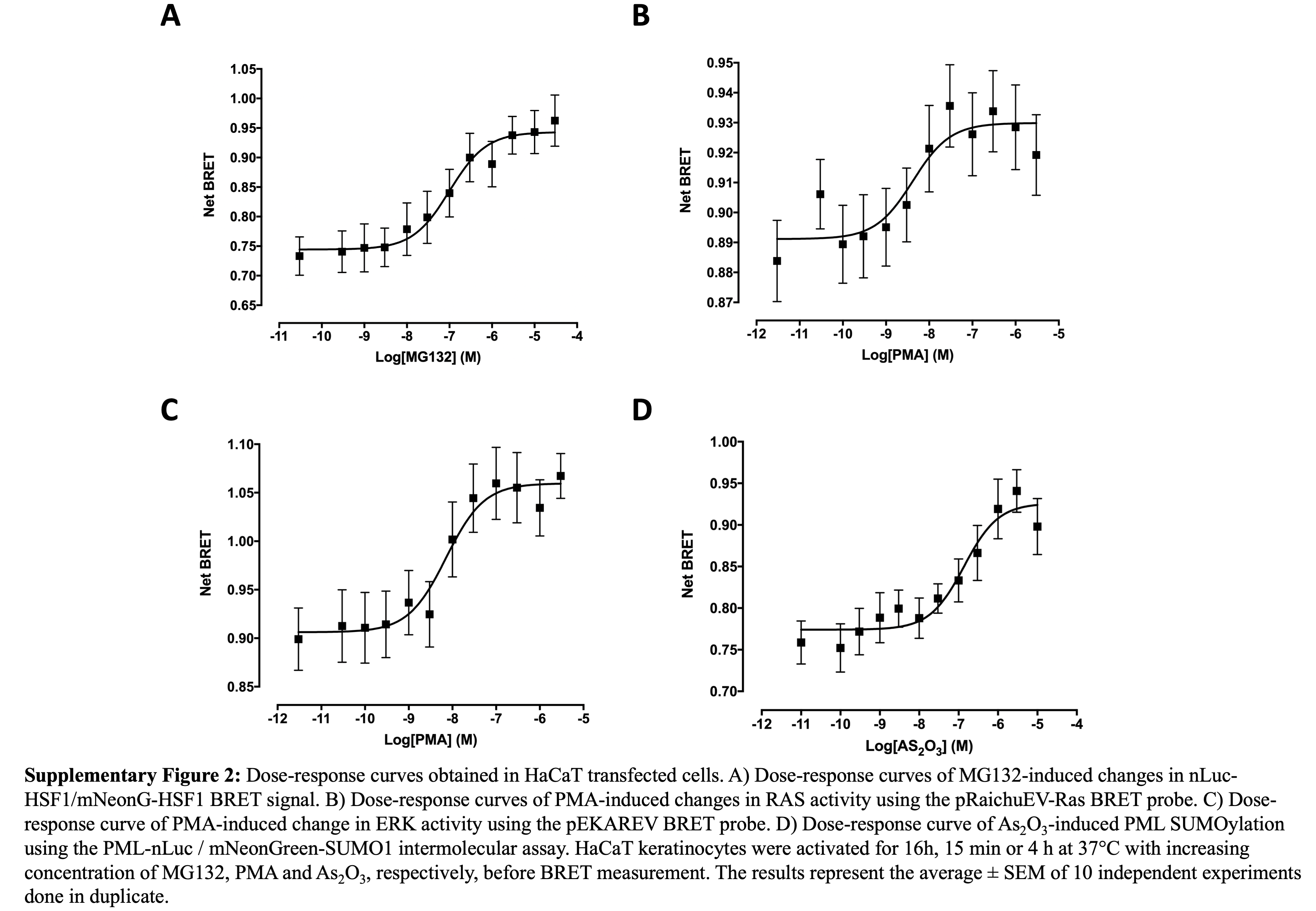

Supplement: Supplementary file 2 — Supplementary Figure 2. [file 41598_2023_35397_MOESM2_ESM.tiff]
